# Effects of auricular acupuncture stimulation on healthy adults’ upper limb motor-evoked potentials: A randomized, crossover, double-blind study

**DOI:** 10.3389/fnins.2022.895602

**Published:** 2022-08-18

**Authors:** Jin Zhang, Wen-Hao Huang, Ya-Dan Zheng, Xin Li, Hao-Xiang Jiang, Min-Zhi Su, Xiao-Yan Huang, Zu-Lin Dou, Zhi-Ming Tang

**Affiliations:** Department of Rehabilitation Medicine, The Third Affiliated Hospital of Sun Yat-sen University, Guangzhou, China

**Keywords:** cerebral motor cortex, auricular acupuncture, transcranial magnetic stimulation, upper limb function, brain excitability

## Abstract

**Objective:**

The aim of this study was to determine whether auricular acupuncture has neuromodulatory effects on the motor cortex of healthy adults.

**Methods:**

Fourteen healthy subjects received a real auricular acupuncture stimulation (SF1) session and a sham acupuncture stimulation session. The interval between the two types of stimulation was more than 24 h. A finger dexterity test (taping score and taping speed by using ipad) was assessed, and motor-evoked potentials (MEP) were assessed before and after each stimulation.

**Results:**

Before the treatment, there were no significant differences in MEP amplitude, tapping score, or tapping speed (*P* > 0.05) between the real and sham stimulation conditions. After the treatment, the MEP amplitude, tapping score, and tapping speed in the real stimulation condition increased significantly compared to the pre-stimulation measurements and were significantly higher than those in the sham stimulation condition (*P* < 0.01). In the sham stimulation condition, the MEP amplitude, tapping score, and tapping speed decreased significantly compared to the pre-stimulation measurements (*P* < 0.05).

**Conclusion:**

Acupuncture of auricular points can modulate the excitability of the motor cortex area of controlling the upper limbs.

**Clinical trial registration:**

[http://www.chictr.org.cn/index.aspx], identifier [ChiCTR2100051608].

## Introduction

The incidence of stroke has been steadily increasing in China in recent years owing to population aging (Guo et al., 2021). The fatality rate of stroke patients is currently decreasing due to the development of thrombolysis, surgery, and pharmacotherapy in the early stages. However, achieving acceptable treatment outcomes remains difficult, and the disability rate remains high (Owen and Corfe, 2017; Zhao et al., 2017). Upper limb dysfunction is a common clinical symptom after a stroke and has a negative impact on patients’ quality of life (Guo et al., 2021).

Traditional Chinese medicine has considerable experience treating stroke-related dysfunction and has the advantages of simplicity and low cost. The efficacy of acupuncture in post-stroke treatment has been demonstrated and recognized by Chinese experts (Zhang X. Y. et al., 2019). A recent clinical study showed that acupuncture may have positive effects on neuroplasticity, neurochemical changes, and the activity of specific brain areas (Liu et al., 2018). Acupuncture of the upper limbs and scalp is increasingly used to treat upper limb motor dysfunction following a stroke. However, needles cannot be used in some situations, such as when there is a skin disease or an incomplete skull after craniectomy.

Auricular acupuncture, a form of microneedle therapy that combines acupuncture and the holographic theory, is a well-accepted therapeutic method that is safe, effective (Litscher and Rong, 2016). Auricular acupuncture has been used to treat stroke patients with hemiplegia (Jian, 2009), depression (Zhang et al., 2017), and hypertension (Abdi et al., 2017). However, previous studies have reported only the clinical effects of auricular acupuncture, while its mechanism of action remains unknown. Moreover, few studies have evaluated the effects of sham auricular acupuncture using control groups. Some researchers have demonstrated that stimulating auricular acupoints decreases heart rate variability and maintains a relatively consistent level of autonomic function (Arai et al., 2013; Litscher and Rong, 2016). However, the international promotion of auricular acupuncture is hampered by a lack of objective evaluation metrics, particularly metrics related to its effects on the central nervous system.

Transcranial magnetic stimulation (TMS) is a non-invasive technique for determining the excitability of the cortical spinal tract that can be used to investigate the effects of acupuncture on the central nervous system (Maioli et al., 2006; Yang et al., 2017; Tang et al., 2019). Variations in the amplitude of motor-evoked potentials (MEP) induced by TMS are frequently used to detect differences in cortical excitability (Du et al., 2018). Numerous recent studies have reported MEP changes in response to acupuncture, but the findings have been inconsistent (Sun et al., 2019; Li et al., 2021). Furthermore, it is unknown whether auricular acupuncture regulates the motor cortex. Therefore, this study utilized TMS to determine the effect of auricular acupuncture on upper limb function and cortical activity.

## Methods

### Subjects

According to the data collected from our preliminary experiment, 14 healthy volunteers (six males and eight females; mean age: 25.29 ± 6.23 years) participated in this study. The inclusion criteria were (1) healthy adults aged between 18 and 60 years, (2) right-handedness according to the Edinburgh Handedness Scale, and (3) no underlying conditions or musculoskeletal injury to the right upper limb. The exclusion criteria were (1) intolerance to magnetic stimulation or acupuncture, (2) allergy to electrode pads, (3) use of a pacemaker or metal implants, (4) incomplete, broken, or infected ears (holes in the earlobe were not considered to constitute a broken ear), and (5) pregnancy or lactation.

The study protocol was approved by the Ethics Committee of the Third Affiliated Hospital of Sun Yat-sen University [(2021)02-257-01]. The study was registered in the Chinese Clinical Trial Registry (ChiCTR2100051608). All participants signed written informed consent forms.

### Study design and procedure

**FIGURE 1:**
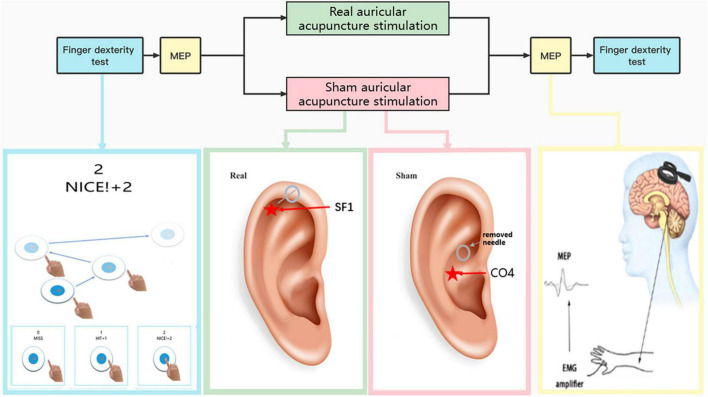
Flowchart of the experiment.

This study followed Standards for Reporting Interventions in Clinical Trials of Acupuncture (STRICTA): extending the consolidated standards of reporting trials (CONSORT) Statement (MacPherson et al., 2010). A randomized, crossover, double-blind design was used in this study. Each participant received a real and a sham acupuncture stimulation session (Figure 1). To eliminate order effects, half of the participants received the real stimulation first and then the sham stimulation, and the other half received the sham stimulation first and then the real stimulation (Table 1). Random orders were generated by using Microsoft Excel, and the orders were secured with a person who did not participate in any of the assessments. To avoid lingering effects, the interval between the two stimulation types was following 24 h. The participants were advised to abstain from any drugs or beverages that could impair brain function during the study. Each stimulation session lasted 5 min.

**TABLE 1:** Subjects’ demographic information and stimulation order.

Subject	Age	Gender	Stimulation order	
			
			First stimulation	Second stimulation
1	25	F	Real	Sham
2	26	M	Real	Sham
3	31	F	Real	Sham
4	33	F	Sham	Real
5	20	M	Real	Sham
6	21	M	Sham	Real
7	24	M	Sham	Real
8	25	M	Real	Sham
9	22	F	Real	Sham
10	23	F	Sham	Real
11	21	F	Real	Sham
12	22	F	Sham	Real
13	19	M	Sham	Real
14	42	F	Sham	Real

F, female; M, male.

### Acupuncture methods

The experiment was conducted by three operators. An acupuncturist stimulated the subjects’ auricular acupoints. Another operator, blinded to the experimental condition, performed TMS to obtain the subjects’ MEPs. A third operator collected data from the finger dexterity tests performed before and after each stimulation (after removing the needles and adhesive tape).

The experiments took place in a tranquil and comfortable setting. Each subject spent the entire time in an armchair, in a relaxed and pleasant sitting position. A pillow was placed on his or her thighs, and his or her hands were resting on the pillow.

Real auricular acupuncture stimulation: The operator disinfected the skin at the left ear point (SF1) and then wiped away the disinfectant with a sterile dry cotton swab (Bullock et al., 2002). The acupoint was pierced with a disposable sterile intradermal needle measuring 0.20 mm× 1.30 mm, which was taped to the skin. The stimulation duration was 5 min.

Sham auricular acupuncture stimulation: We selected CO_4_ as sham auricular acupoint (Figure 1). We removed the tip of needle and only retained the circular needle handle. The circular needle handle was taped to the skin of CO_4_. The stimulation duration was 5 min (Tan et al., 2015; Jan et al., 2017; Ots et al., 2020).

### Transcranial magnetic stimulation

Each participant was comfortably seated in a tall chair, with the forearms, back, and legs supported. The procedure was then explained and demonstrated to the participant. The skin of the participant’s index finger was cleaned and prepared to achieve a low skin impedance of ≤10 kΩ. A pair of chloride-surface electromyography (EMG) electrodes was then placed on the first dorsal interosseous muscle of the dominant hand. Grounding electrodes were placed on the ipsilateral ulnar styloid process. A surface electromyography instrument (Neuron-Spectrum-5, Neurosoft, Russia) recorded the MEP with the filtering set to 5–10,000 Hz, sampling rate 25,000 Hz, the scanning speed set to 5 ms/div.

During the TMS measurements, the participant was wearing a nylon cap. The position of the C3 was marked as a reference point for localizing representative areas of the hand in the primary motor cortex according to the electroencephalogram (EEG) International 10–20 system. A TMS system (YD-MT500, Neurosoft, Ivanovo, Russia) and a 100-mm figure-of-eight coil, with the handle of the coil facing 45° behind the center line, were used. The area around the marked C3 point was initially stimulated using 65% of the TMS instrument’s maximum output intensity. The most excitable site in the primary motor cortex consistently eliciting large MEP amplitudes was marked as the “hot spot.”

Once the participant’s hotspot was identified, the rest motor threshold (rMT) was determined by gradually reducing the suprathreshold TMS output intensity initially by 2% and then by 1% as the threshold intensity was approached. The rMT was defined as the lowest output intensity that could produce an MEP amplitude of at least 50 μV for the first interosseous muscle in at least 5 out of 10 consecutive TMS stimulations.

The participant subsequently rested for 1 min and was then measured at an output intensity of 120% rMT to obtain the peak MEP amplitude. Five measurements were performed (Tang et al., 2019), with rest intervals of 5–10 s between them, and the average was calculated.

### Finger dexterity test

A finger dexterity test was performed to evaluate the participants’ simple response times using an iPad app developed by Masaharu Tsukamoto.^1^ Over a 30-s period, the participants rapidly taped on randomly appearing circles on the screen. This test was performed before and after each stimulation (Figure 1).

Two kinds of test results were obtained: tapping score and tapping speed (tapping times/second). To obtain the tapping score, the subjects were instructed to tap on the center of the target circle as fast as possible. Circular patterns appeared randomly on any part of the screen. A score of 2 meant that the subject taped on the center of the circle. A score of 1 meant that the subject taped on the inner part of the circle but not on the center. A score of 0 meant that the subject taped on an area outside the circle.

### Data and statistical analyses

Using the G*Power statistical tool version 3.1, it was determined that a sample size was required to achieve a statistical power of 80% with statistical significance at *P* < 0.05 (two-tailed) based on preliminary experimental results. All statistical analyses were performed using SPSS 22.0. Data normality and homogeneity of variance were examined using the Shapiro–Wilk test. All variables were normally distributed and represented as means ± standard deviations. Paired *t*-tests were used to assess differences before and after the stimulation sessions and between the real and sham conditions in terms of peak MEP amplitude, tapping score, and tapping speed. Values of *P* < 0.05 indicated statistically significant differences.

## Results

### Motor-evoked potentials amplitude

**FIGURE 2:**
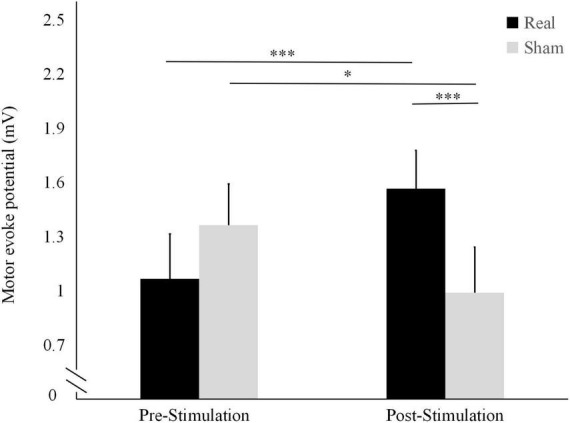
Differences in motor-evoked potentials (MEP) amplitude before and after the real and sham stimulation sessions. ***P < 0.001, *P < 0.05.

As shown in Figure 2, the mean MEP was significantly higher after the stimulation (1.56 ± 0.55 mV) than before the stimulation (1.06 ± 0.46 mV; *t* = 4.564, *P* < 0.001) in the real condition. Conversely, it was significantly lower after the stimulation (1.01 ± 0.22 mV) than before the stimulation (1.31 ± 0.51 mV; *t* = -2.562, *P* = 0.024) in the sham condition.

The pre-stimulation MEP did not differ significantly between the two conditions (*t* = 1.712, *P* = 0.111). Conversely, the post-stimulation MEP was significantly higher in the real stimulation condition than in the sham condition (*t* = 4.256, *P* < 0.001).

### Finger dexterity test

**FIGURE 3:**
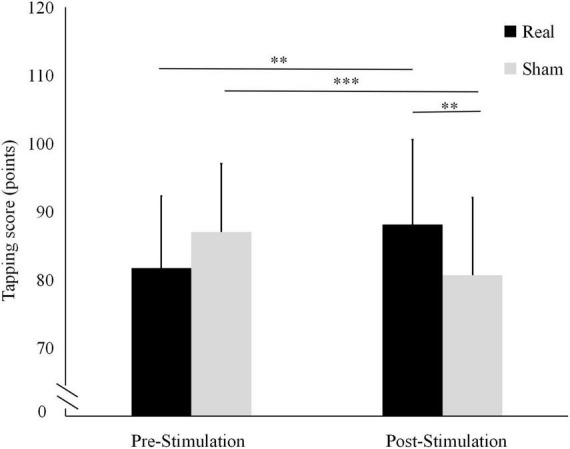
Differences in tapping scores before and after the real and sham stimulation sessions. ***P < 0.001, **P < 0.01.

The tapping score results are shown in Figure 3. In the real stimulation condition, the mean tapping score was significantly higher after the stimulation (88.14 ± 12.46) than before the stimulation (81.71 ± 10.6; *t* = 3.019, *P* = 0.010). Conversely, in the sham condition, it was significantly lower after the stimulation (80.71 ± 11.38) than before stimulation (87 ± 10.05; *t* = −5.840, *P* < 0.001).

**FIGURE 4:**
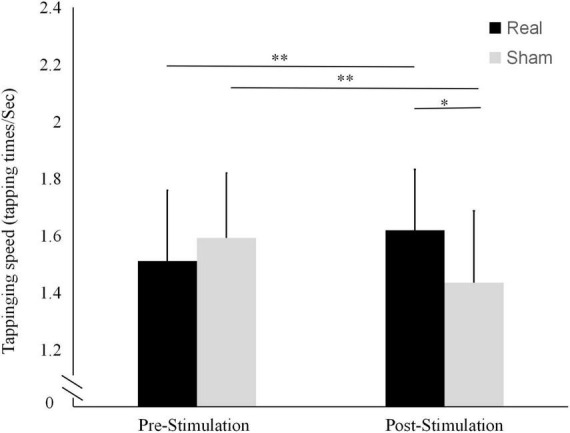
Differences in tapping frequencies before and after the real and sham stimulation sessions. **P < 0.01, *P < 0.05.

The results of tapping speed are shown in Figure 4. In the real stimulation condition, the mean tapping speed was significantly higher after the stimulation (1.62 ± 0.21 tapping times/sec) than before the stimulation (1.51 ± 0.25 tapping times/sec; *t* = 3.362, *P* = 0.005). In contrast, in the sham condition, it was significantly lower after the stimulation (1.44 ± 0.25 tapping times/sec) than before the stimulation (1.59 ± 0.22 tapping times/sec; *t* = -3.435, *P* = 0.004).

There were no statistically significant differences between the two conditions in terms of pre-stimulation tapping score (*t* = -1.949, *P* = 0.073) or tapping speed (*t* = -1.700, *P* = 0.113). Conversely, both the tapping score and the tapping speed after the stimulation were significantly higher in the real stimulation condition than in the sham condition (*t* = 3.315, *P* = 0.008 and *t* = 2.863, *P* = 0.013, respectively).

## Discussion

Auricular acupuncture, a form of traditional Chinese medicine, is frequently used at stroke rehabilitation centers (Jian, 2009; Litscher and Rong, 2016). However, the mechanism by which auricular acupuncture affects the central nervous system is unknown. This study examined whether the stimulation of auricular acupoints can alter cortical excitability and motor function, which might suggest that the therapeutic effects of auricular acupuncture are related to central nervous system modulation.

The observed increase in MEP amplitude after the real stimulation suggests that auricular acupuncture can significantly enhance cerebral motor cortex excitability. Conversely, the reduced MEP amplitude after the sham stimulation could be because in the absence of brain stimulation, the 5-min resting state induced a sedative state in the brain.

The tapping test results suggest that needling the auricular area improves finger dexterity. The tapping score, which represents the speed and accuracy of tapping, can be compared to the rules of archery, while the tapping times/sec only represent the speed of finger movement. Linking the behavioral data to the corticospinal excitability data provided a greater degree of certainty about the role of auricular acupuncture in cortical excitatory changes (Maioli et al., 2006; Liu et al., 2018).

The changes in MEP amplitude and tapping performance suggest an ear–brain–upper limb interaction, in which input is routed to the brain *via* the corticospinal tract. A previous study performing acupuncture stimulation of 20 healthy individuals’ ST36 acupoints, located on the lower limb, reported a limb–brain interaction. The subjects’ cortical excitability was significantly higher after the stimulation compared to a sham stimulation condition (Sun et al., 2019). The reorganization of motor neuron pools could be a result of the “Deqi” sensation (Liang et al., 2013; Yuan et al., 2013). Acupuncture stimulation has been shown to exert physiological effects on the autonomic nervous system by activating a somatosensory pathway (Kim et al., 2012; Yang et al., 2017). The ear–brain–upper limb interaction suggested by our experimental results explains how the stimulation of ear acupoints can alter cortical excitability.

A previous study reported that acupuncture in the hepatobiliary region of rabbits’ ears increased the availability of synaptic dopamine, which may play a critical role in motor function improvement (Li et al., 1992). In that investigation, stimulation from the ear vagus branch traveled *via* the vagus nerve to the medulla oblongata, establishing an ear–brainstem connection. Several other studies using electric stimulation or TMS to stimulate the vagus have suggested that vagus fibers are distributed in the ear (Fang et al., 2016; Zhang Y. et al., 2019; Luo et al., 2020; Wang et al., 2021; Zhang et al., 2021). Auricular acupuncture may influence the central nervous system through sensory circuitry, enhancing the excitability of the motor cortex through the sensory cortex. However, none of these studies has conclusively established a link between the ear and the motor region of the brain. Our study is the first to establish a connection between the ear and the motor areas of the brain using MEPs manifested in the limbs. The Delta reflex theory postulates a bidirectional reflex somatic (visceral)–brain–ear pathway (Jing-xuan et al., 2021). Our findings suggest a limb–brain–ear association with an additional pathway associated with the corticospinal tract. This pathway is quite similar to the one proposed by the Delta reflex theory and should be further investigated.

In terms of methodology, we used the MEP amplitude as the primary objective indicator to determine whether auricular acupuncture can modulate cerebral cortex excitability. We also employed sham stimulation to eliminate the possibility of an auricular acupuncture placebo effect. Moreover, we employed a self-controlled study design with two conditions for all subjects, which can reduce the necessary sample size and individual variations. Furthermore, we set 24-h intervals between the sham and real auricular acupuncture sessions to ensure retention effect washout and reliable comparisons between the two conditions in terms of tapping score, tapping speed, and MEP.

Our study has several limitations. Firstly, our experiment could only demonstrate that auricular acupuncture applied to SF1 could result in M1 excitation. It is necessary to investigate whether it causes excitation in other areas of the brain or whether acupuncture of other auricular points can cause excitation in the M1 area. Secondly, we did not establish clear guidelines for the strength, direction, and depth of ear point needling. Third, we needled only the left ear and did not compare the two ears or the dominant and non-dominant hands. Finally, we only measured the peak MEP amplitude to assessed corticospinal tract excitability, however, the pathway from the ear to the corticospinal remains unknown. In subsequent research, the experimental design could be refined, and alternative observation methods may be used to overcome these limitations.

## Conclusion

Acupuncture of auricular points can modulate the excitability of the motor cortex area of controlling the upper limbs and improving the finger motor function.

## Data availability statement

The original contributions presented in this study are included in the article/supplementary material, further inquiries can be directed to the corresponding authors.

## Ethics statement

The studies involving human participants were reviewed and approved by the Ethics Committee of The Third Affiliated Hospital of Sun Yat-sen University. The patients/participants provided their written informed consent to participate in this study. The study was registered in the Chinese Clinical Trial Registration (ChiCTR2100051608).

## Author contributions

JZ and W-HH took responsibility for the integrity of the data and the accuracy of the data analysis. Z-LD and Y-DZ contributed to data analysis and data acquisition. XL, H-XJ, M-ZS, and X-YH made critical revision of the manuscript for important intellectual content. Z-MT guided the research and revised the manuscript. All authors contributed to the conception, design, analysis, and interpretation of data of the study.

## Footnotes

1
https://apps.apple.com/cn/app/id1141314478
